# The evolutionary origin of avian facial bristles and the likely role of rictal bristles in feeding ecology

**DOI:** 10.1038/s41598-022-24781-7

**Published:** 2022-12-06

**Authors:** Mariane G. Delaunay, Charlotte Brassey, Carl Larsen, Huw Lloyd, Robyn A. Grant

**Affiliations:** 1 Department of Natural Sciences, Manchester Metropolitan University, Manchester, M1 5GD UK; 2 School of Life Sciences, University of Liverpool, Liverpool, UK

**Keywords:** Behavioural ecology, Evolutionary ecology, Animal behaviour, Animal physiology

## Abstract

Facial bristles are one of the least described feather types and have not yet been systematically studied across phylogenetically diverse avian species. Consequently, little is known about their form, function and evolutionary history. Here we address this knowledge gap by characterising the evolution of facial bristles for the first time. We especially focus on rictal bristle presence and their associations with foraging behaviour, diet and habitat preferences in 1022 avian species, representing 91 families in 29 orders. Results reveal that upper rictal, lower rictal and interramal bristles were likely to be present in the most recent common ancestor of this avian phylogeny, whereas narial bristles were likely to be absent. Rictal bristle presence, length and shape varied both within and between avian orders, families and genera. Rictal bristles were gained or lost multiple times throughout evolution, which suggest that the different morphologies observed within species might not be homologous. Phylogenetic relatedness is also not likely to be the only driver of rictal bristle presence and morphology. Rictal bristle presence and length were associated with species-specific ecological traits, especially nocturnality. Our findings suggest that species foraging in low-light conditions are likely to have longer rictal bristles, and that rictal bristles are likely to have evolved in early birds.

## Introduction

**Figure 1:**
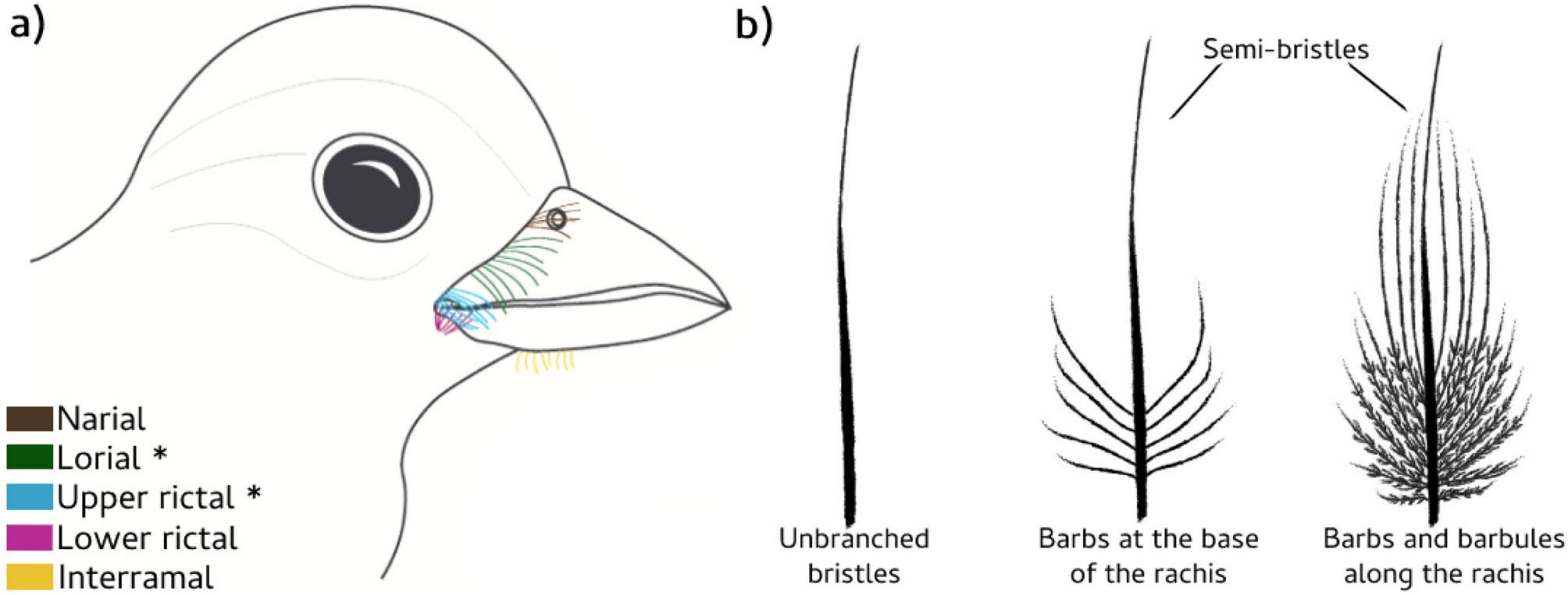
Diagram illustrating the location and shape of facial bristles. (**a**) Bristle locations: narial, lorial, upper rictal, lower rictal and interramal bristles; (**b**) bristle shapes: unbranched bristles, and bristles (semi-bristle type) with barbs only at the base, and with barbs and barbules along the rachis. Asterisks (*) indicate the two bristle locations that have been grouped as “rictal bristles” in this study.

Birds have undergone many significant morphological changes throughout their evolution^1^, leading to a great diversity of traits^2^. Feathers are perhaps one of the most complex and diverse morphological structures in birds^3,4^, with each type (flight feathers, down feathers, filoplumes, bristles etc.) varying in shape, size and function^5,6^. Correspondingly, in modern-day birds, facial bristles, which are present on the rictal, lorial, narial and interramal regions, also exhibit diverse morphological characteristics (Fig. 1). They can be unbranched, resembling hair-like structures (true bristles), or they can have branching barbs or barbules (i.e. semi-bristles)^7,8^ (Fig. 1a). However, facial bristles are one of the least described feathers, and their presence has not yet been extensively and systematically recorded across species. Consequently, their origin and evolution within birds are unknown. The genetic pathway of rictal bristles has also yet to be investigated. Therefore, it is uncertain whether the different morphologies of rictal bristles represent a homologous structure evolved from a common ancestor.

Of all facial bristles, the rictal bristles (which we group as both the upper rictal and lorial bristles, Fig. 1a), are perhaps the best described. Even so, rictal bristle morphology has only been described in a small number of endemic New Zealand bird species^9^, as well as New World Alder flycatchers^10^, and several nightjar species and their relatives^11^. In these species, innervation and mechanoreceptors (Herbst corpuscles) have been found to be present around the rictal bristle follicles of a small number of the species examined, but also absent in others^9,11^. These findings indicate that bristles may play a vibrotactile role, which may aid in nocturnal navigation, foraging and burrow nesting in some avian species (e.g. kiwis)^9,11,12^, as well as providing protection to the eyes from food parts and flying particles^10^. Delaunay et al.^11^ found that nocturnal cathemeral Caprimulgiformes species that forage in open habitats had less sensitive rictal bristles, suggesting that rictal bristles may be associated with nocturnal foraging in closed habitats. The functional implications of early-feathers in fossil birds is also unknown, and a better understanding of rictal bristles in modern birds might help us gain insights into the evolution and function of this trait.

Indeed, despite being present in many nocturnal and diurnal species, knowledge about the exact functional role of rictal bristles is still unknown as previous studies have been phylogenetically constrained, and only examined in species in closely-related groups. Therefore, an examination of bristle presence and form across a larger range of avian species would give us greater insights into the evolution and function of avian bristle touch sensing. In this study we describe the presence of facial bristles in 1022 avian species (~ 10% of all recorded species), including 418 genera, from 91 families (37% of all recorded families) and 29 orders (73% of all recorded orders). We document the evolution of different facial bristles around the beak, using ancestral state reconstruction analysis visualised by stochastic character state mapping. Since rictal bristles are likely to be involved in foraging in low-light, closed habitats^11^, we go on to examine the function of rictal bristles by investigating their association with a range of ecological traits, including period of activity, habitat type, foraging method, foraging height and diet.

## Results

### Evolution of facial bristle presence

**Figure 2:**
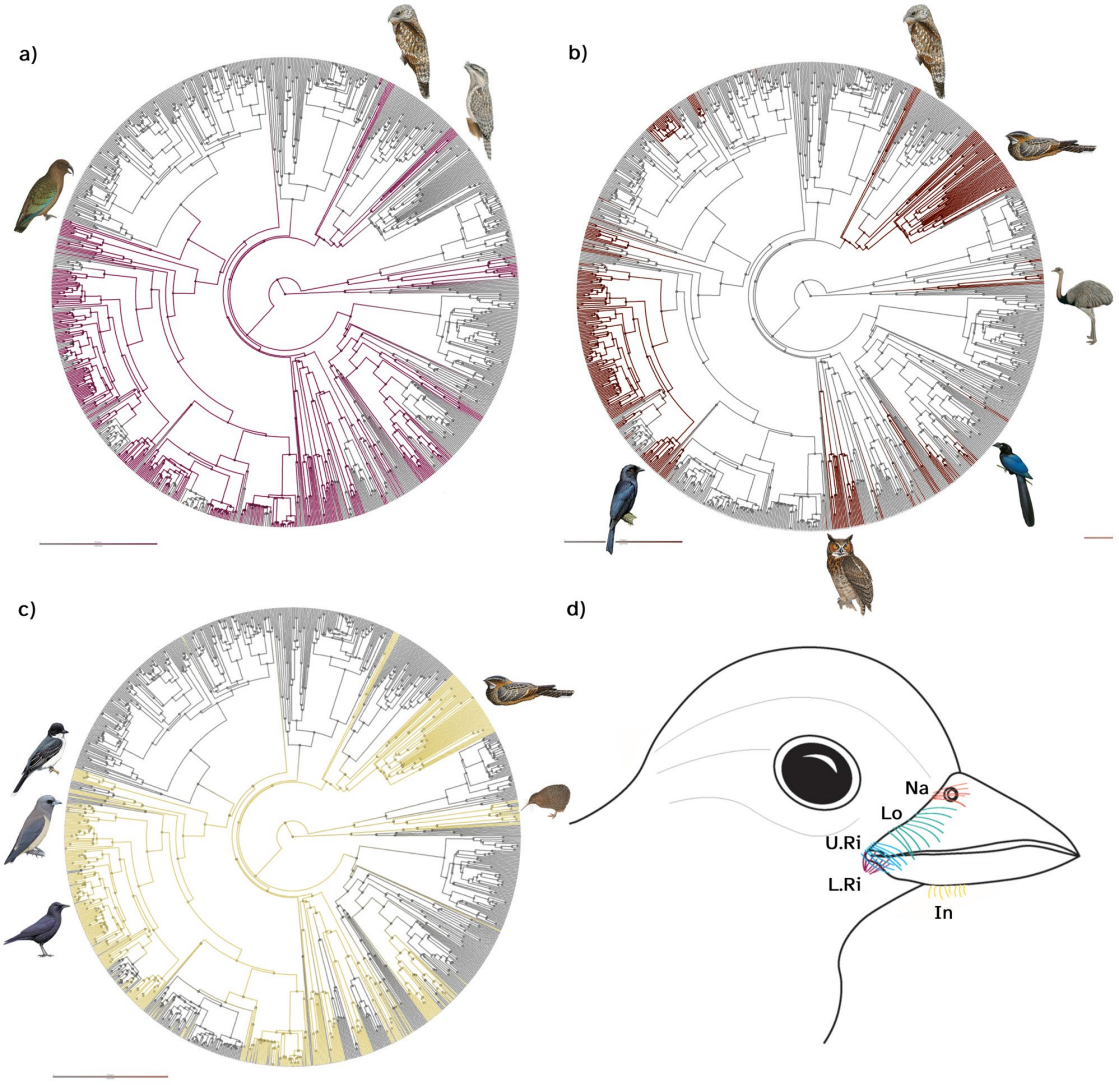
Phylogenetic trees mapping the ancestral character estimation for the presence of facial bristles at different locations: (**a**) lower rictal, (**b**) narial, (**c**) interramal bristles; (**d**) schematic drawing of a bird head illustrating the position of the different types of facial bristles around the beak (Na: narial, Lo: lorial, U.Ri: upper rictal, L.Ri: lower rictal, In: interramal bristles). Terminal branches of the phylogeny correspond with the different species measured. The colour of the branches in the tree gives the posterior probability of the facial bristle character through avian evolution; grey indicates the high probability of the different facial bristles being absent while other colours indicate the presence of these facial bristles.

Lower rictal and interramal bristles were most likely present in the ancestor of the species measured in our dataset (100% and 97% confidence in ASR, respectively), whereas narial bristles were likely absent (98% confidence in ASR; Fig. 2a–c, Table S1). Each facial bristle location had a strong significant phylogenetic signal (Table S1; *phylosig*, λ > 0.70, *P* < 0.001). Stochastic character state mapping reported multiple changes between states across the avian phylogeny, of which a higher number of changes were in favour of a gain in narial bristles, and a loss in lower rictal and interramal bristles (Table S1). Most species with rictal bristles also had bristles on the narial (67%, n = 245) and on the interramal (79%, n = 289) regions (Fig. 2). Bristles on the lower rictus were predominant in 55% of all Passeriformes species measured, as well as in all Strigopidae, Strigiformes, Trogoniformes, Momotidae and Numididae species, and in some Coraciidae (71%, n = 14), Cuculidae (32%, n = 133) and Cracidae (36%, n = 14). Most Caprimulgimorphae species did not present any bristles on the lower rictus, with the noticeable exceptions of Podargidae and Nyctibiidae (Fig. 2).

**Figure 3:**
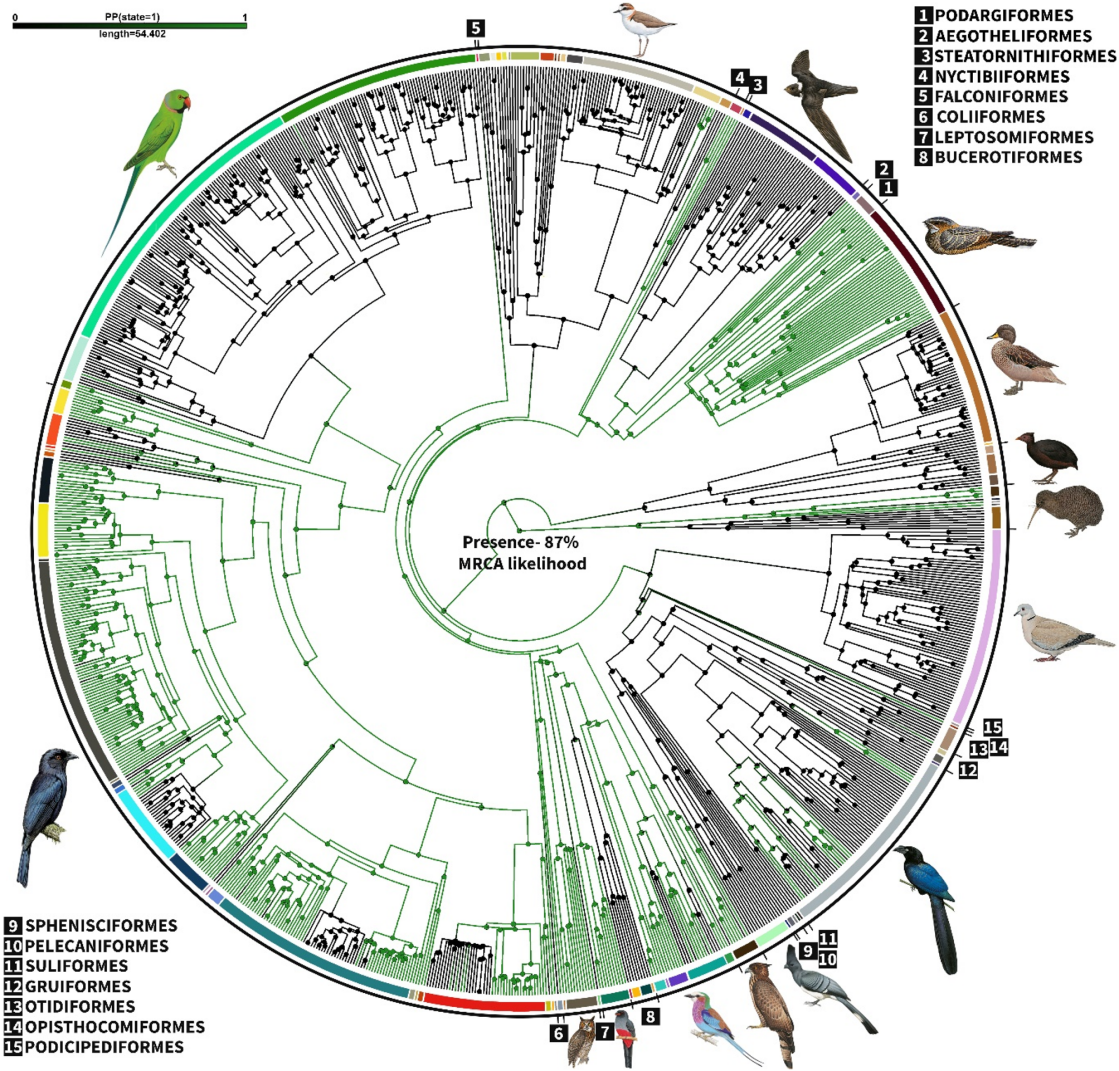
Phylogenetic trees mapping the ancestral character estimation for the presence of rictal bristles. Terminal branches correspond with the different species measured. The colour of the branches in the tree gives the posterior probability of the rictal bristle character through avian evolution; black indicates a high probability of rictal bristle absence while green indicates the presence of rictal bristles. The inner coloured arc encircling the radial phylogenetic tree illustrates the different avian families to which each species belongs, and the black outer circle corresponds to avian orders.

Our ancestral character state reconstruction analysis revealed that the presence of rictal bristles (defined as including both lorial and upper rictal bristles; Fig. 1) was the most likely ancestral state, with an 87% likelihood of being reconstructed at the basal node of the phylogeny (Fig. 3). Rictal bristle presence had a significant phylogenetic signal (*phylosig*, λ = 0.89, *P* < 0.001) and were most notably present in Palaeognathae species (e.g. Apterygidae and Rheidae), Caprimulgimorphae (e.g. Caprimulgidae, Podargidae and Aegothelidae) and Passeriformes (e.g. Corvidae, Oriolidae, Artamidae and Dicruridae, Fig. 3). Stochastic character mapping reported an average of 63 changes between character states across the avian phylogeny, with an average of 16 state changes in favour of rictal bristle gain and an average of 47 state changes towards a loss (Fig. 3).

### Rictal bristle morphology

No significant difference was found between the presence of rictal bristles on males or female species. There was also no significant difference in rictal bristle length (Mann–Whitney U test, W = 37,962, N = 552, *P* = 0.94) and shape (Chi-square test, χ^2^ = 0, N = 552, df = 3, *P* = 1) between males and females either. However, bristle length varied greatly within species, ranging from 0 (when absent) to 41.38 mm (in *Antrostomus sericocaudatus*) when taken per individual, or ranging from 0 to 39 mm (*Steatornis caripensis*) when averaged per species. Similarly, bristle shape varied amongst order, family and species. 657 species did not have rictal bristles (absent in 61 families and 23 orders), 26 species had bristles with barbs at the base (including 10 family and 6 orders), 47 species had bristles branched along the rachis (including 13 families and 9 orders) and 292 species had unbranched bristles (including 33 families and 13 orders).

### Association of rictal bristles with ecological traits

**Figure 4:**
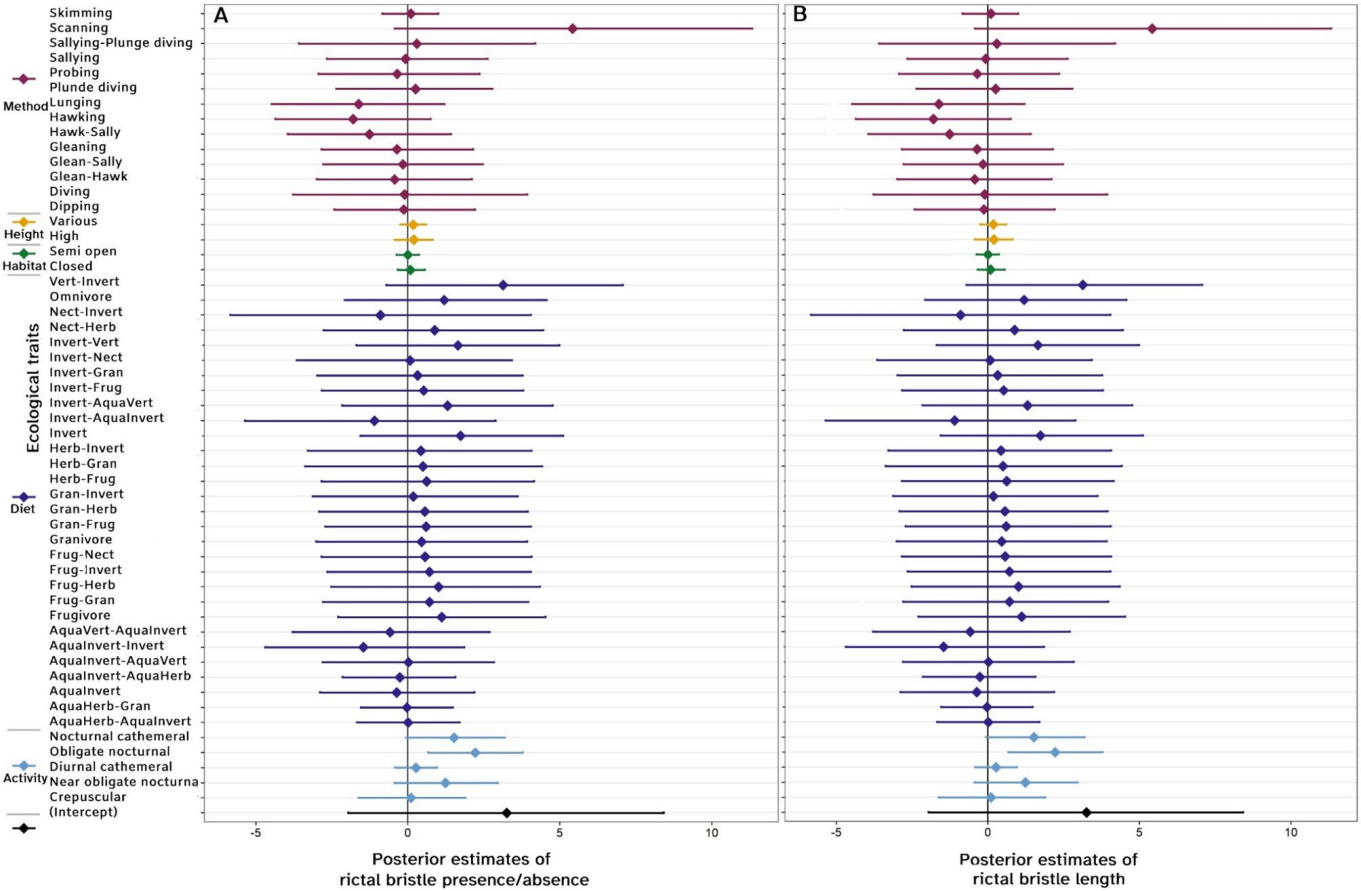
Caterpillar plots representing the posterior mean estimates and the 95% credible intervals for each ecological predictor in models, rictal bristle presence and length models. The colours of the bars represents the different ecological traits: activity period in blue, diet in dark purple, habitat type in green, foraging height in yellow, foraging method in dark magenta, and the model intercept in black.

Period of activity significantly predicted the presence of rictal bristles. Specifically, crepuscular (*p*MCMC = 0.01), near obligate nocturnal species (*p*MCMC = 0.036) and obligate nocturnal species (*p*MCMC = 0.006) were more likely to have rictal bristles than diurnal species (Fig. 4). By contrast, diet, foraging height and habitat type did not significantly predict rictal bristle presence (Fig. 4).

Foraging method was a significant predictor of the rictal bristle length. Specifically, rictal bristles were longer in species that are obligate nocturnal (*p*MCMC = 0.005; Fig. 4) compared to diurnal. Other foraging methods, period of activity, foraging height, habitat type and diet were not significant predictors of rictal bristle length in our model (Fig. 4).

## Discussion

In our study, we describe for the first time, the presence of facial bristles throughout an avian phylogeny and its most recent common ancestor (MRCA). We show that rictal bristles were present in 35% of the species we measured in this study, and were likely to be ancestral. The prevalence and diversity of facial bristles across our phylogeny suggest that they are likely to be functional, despite being relatively understudied in the past compared to other avian senses.

Our study reveals that rictal bristles are more common than originally thought and may be present as an ancestral state in our phylogeny. Indeed, the MRCA of this phylogeny (~ 108 mya) was likely to have bristles on the rictal, lower rictal and interramal regions, but not on the narial region. Previous studies have not yet considered the evolution of bristles. Chen et al.^13^ incorporated bristle presence into a study of many morphological characteristics in Strisores, and assumed that bristles were a shared derived trait of Caprimulgimorphae. We, rather, suggest that rictal bristles were retained in Caprimulgimorphae and might be an ancestral state, derived from an earlier ancestor, common to Palaeognathae, Caprimulgimorphae and other Neoaves.

All avian feathers are diverse^5,6^ and our study suggests that rictal bristles are no exception. They exhibit pronounced variation in length, shape, and position across species, within the same orders, families and genera (i.e. Caprimulgimorphae and Passeriformes, Fig. 1). For instance, Apterygidae have many different types of facial bristles. They have all the types described in this study, as well as orbit and forehead bristles^9^. They are also are well known for having long bristles (19 to 37 mm average per individual, across species) that are unbranched or with barbs at the base. Within our dataset, the oilbird species (*Steatornis caripensis*) had the longest unbranched rictal bristles (39 mm) followed by the silky-tailed nightjar species (*Antrostomus sericocaudatus*; unbranched, 38 mm). The presence of many events of bristle disappearance and, especially re-apparitions, in addition to their diverse morphologies, may suggest that rictal bristles are not a homologous structure. Nevertheless, Delaunay et al.^11^ and Cunningham et al.^9^ found very similar follicle anatomy in both bristles and semi-bristles type of facial bristles, in Caprimulgimorphae and some New Zealand bird species. While we suggest that facial (rictal, lower rictal and interramal bristles) bristles are ancestral, it is worth bearing in mind that the genetic pathways of rictal bristles have not yet been investigated. Therefore, it is not possible to infer whether bristles are truly homologous structures. Findings from an investigation into bristle homology may well impact our results on the ancestral state of bristles.

Despite facial bristle presence having a strong phylogenetic signal (Table S1, all *P* < 0.05), phylogeny alone did not explain rictal bristle presence, therefore ecological traits might also be important. While nocturnality is associated with the presence of rictal bristles, obligate nocturnality is especially associated with species having long rictal bristles in our data (Fig. 4; e.g. the oilbird, *Steatornis caripensis*). In contrast, diet, habitat type and foraging height were not significant predictors of rictal bristle presence and length in any of our models. In agreement, Delaunay et al.^11^ found that species within the Caprimulgimorphae that were nocturnal cathemeral tended to have shorter, branched rictal bristles that lacked mechanoreceptors around their follicles compared to nocturnal species. Nocturnal brown kiwis (*Apteryx mantelli*) and moreporks (*Ninox novaeseelandiae*) also possess functional tactile rictal bristles to some degree, since mechanoreceptors are known to be present around the follicles^9^. However, mechanoreceptors are also present around the follicles of the rictal bristles of the diurnal stitchbird (*Notiomystis cincta*), South Island robin (*Petroica australis*) and New Zealand fantail (*Rhipidura fuliginosa*)^9,14^, suggesting that nocturnality is not the only predictor of bristle presence and functionality. Facial bristles may also play a role in foraging, including in prey handling, sensing while foraging on the wing (aerial foraging), orienting in dark environments (e.g. nest cavities), and prey detection^9,15^. Bristles may also represent “hygienic structures”, shielding the nares and eyes from dust, vegetation and food items^9,16^ or from dirt during nest excavation. Persons and Currie^17^ suggested that first feathers were likely bristles, with a tactile function. Therefore, the somatosensory function of rictal bristles may have been retained, or lost, throughout avian evolution. Further investigations into how the bristles are used in a bird’s natural environment will give us greater insights into bristle function, but this remains particularly challenging to document since facial bristles are small and the species are often nocturnal.

### Limitations

While we have traced the evolution of facial bristles, and especially rictal bristles in a large selection of avian species, their functional associations still remain relatively unclear. While we examined a large number of different species, our data only represented 10% of all known species. Further data collection of the presence and morphology of facial bristles from a greater number of species would be beneficial to reconstruct an even earlier ancestral state, as well as to better understand facial bristle evolutionary events (i.e. loss and gains), which in turn could give a better insight into their functional role. The performance of our models could also have an effect on our findings. Models testing predictors of rictal bristle presence and length were generally inconclusive with the exception of nocturnality, which appeared to be a clear predictor of the presence and length of rictal bristles. This may be attributed to the number and selection of species within the models, or alternatively, by the use of broadly defined ecological categories. For instance, habitat type broad categories could be refined by inclusion of elevation or micro-habitat usage (foraging habitat vs roosting habitat vs habitat for nidification). Foraging methods were also challenging to categorise, and there may be some similarity in different categories but which are reported under different names, e.g. sallying vs hawking^18^. Nevertheless, these categories still allowed us to demonstrate that foraging trait may well be a significant predictor of the presence and length of rictal bristles. Additionally, model performance could be improved through the inclusion of increased sampling of other species groups, such as Passeriformes, Falconiformes, Otidiformes and Strigidae, which were less sampled than some other groups. For instance, this study revealed variation in the presence and morphology of rictal bristles within the Passeriformes order (Figs. 3, 4), even when only 5% of the order was sampled here. A more detailed examination of the Passeriformes order, which is diverse and species-rich, might be beneficial to further understand rictal bristle evolution and function.

## Conclusion

Facial bristles are prominent facial sensors displaying morphological diversity across the many avian species in which they are present. Indeed, rictal bristles are present in around one third of modern bird species and can vary in length (0.6–149 mm) and shape (unbranched, barbs at the base and branched along the rachis). Our findings suggest that early birds may well have had rictal, lower rictal and interramal bristles. Further investigations into these facial bristles will give us better insights into avian evolution and sensory ecology, especially in nocturnal birds. Despite their prominence and prevalence, facial bristles have been largely overlooked in previous studies and their function remains poorly understood. We suggest that future studies need to apply a range of functional morphology tools, including phylogenetics, morphometrics and kinematics, in order to illuminate this important avian sense and its role in the radiation of modern birds. Finally, investigating the genetic pathways of rictal bristles will truly confirm if they are homologous structures, and is needed to further explore the evolution of facial bristle morphology.

## Methods

### Samples

We examined 1,022 avian species (~ 10% recorded species) in this study, representing 418 genera, from 91 families (37% recorded families) and 29 orders (73% of all orders). Specimens were from the skin collection of the World Museum Liverpool, Tring Natural History Museum, Manchester Museum and Wollaton Hall Museum, all situated in the United Kingdom. All work was carried out in accordance with ethical regulations at Manchester Metropolitan University and with the permission of all aforementioned museums. Only the best-preserved adult specimens (no signs of cut off feathers or holes in the skin near the beak) were chosen for this study to ensure accurate measurements of bristle length, shape and presence, which should not be affected by the process of skin removal and specimen conservation. Species were randomly chosen, without targeting our sampling towards species known *a prior*i to have bristles. Where possible, two specimens per species were measured (occurring in 82% of all species examined). Specimens of each sex were measured when present; however, this was not always possible since labelling was often inaccurate or missing. In total, the sample included 508 males, 412 females and 374 individuals of unknown sex. Both sexes were examined in 274 species and there was no difference whatsoever between the presence of bristles on male or female species (n = 97 with bristles present and n = 180 with bristles absent for both males and females). Length (Mann–Whitney U test, W = 37,962, N = 552, *P* = 0.94) and shape (Chi-square test, χ^2^ = 0, N = 552, df = 3, *P* = 1) of rictal bristles also did not significantly differ between males and females. Therefore, rictal bristles are likely to be sexually monomorphic and data for males and females was pooled for further analyses. Overall, rictal bristles were absent in 64% of species examined (n = 656) and just over a third of species (n = 366) had bristles present.

### Bristle descriptions

Facial bristles were initially identified by sight and touch in each specimen. Bristles were recorded as either present or absent from the upper rictal, lorial, lower rictal, narial and interramal regions (Fig. 1a). We use the term ‘rictal bristle’ here for bristles on both the upper rictal and/or the lorial region, since there was no clear differentiation and morphological differences between the bristles found in these regions forming a continuum of bristles above the edge of the beak. When present, rictal bristle shape was recorded as: (i) unbranched rictal bristles, (ii) rictal bristles with barbs only at the base (“Base”) and (iii) branched rictal bristles (“Branched”), i.e. barbs and barbules present along the bristle rachis (Fig. 1b). The three longest rictal bristles were measured on both sides of the head of each specimen using digital callipers, and these lengths were averaged to provide a mean length of rictal bristles per species. In species lacking rictal bristles, a length of “0” and a shape category of “Absent” was recorded.

### Ancestral reconstruction of facial bristle presence

Following Felice et al.^19^, a single consensus phylogenetic tree was generated from the Hackett posterior distribution of trees from Birdtree.org^20^ with a sample size of 10,000 post burn-in, using the TreeAnnotator utility in BEAST software^21^ with a burn-in of 0. Maximum Clade Credibility (MCC) with the option “*-heights ca*” was selected as the method of reconstruction. The common ancestor trees option (*-heights ca*) builds a consensus tree by summarising clade ages across all posterior trees. Both the consensus tree and posterior distribution of 10,000 trees were imported into RStudio v. 1.2.5 for R^22,23^ and pruned so that only species present in the dataset of this study remained in the phylogeny. Taxon names were modified where necessary to match those from the Birdtree.org (http://birdtree.org) species record. Negative terminal branches in our consensus tree were slightly lengthened to be positive using ‘edge.length[tree$edge.length < 0] = 1e−6’. Then, we coerced the tree to be ultrametric using the method ‘nnls.tree’ (phangorn package)^24^ in the function *force.ultrametric* (package phytools v0.7–70)^25^. The root age of the basal node of the consensus tree was calculated using the function *tree.max* from the FossilSim package^26^.

The ancestral state reconstruction (ASR) of the presence/absence character was conducted using the function *make.simmap* (package phytools v0.7-70)^25^, which simulated stochastic character mapping by using the binary character presence/absence of rictal bristles on the consensus tree (nsim = 10,000), with the results summarised by using the function *describe.simmap* (phytools). Three commonly-used evolutionary transition rate models—equal-rates (ER), symmetrical (SYM) and all-rates-different (ARD)—were evaluated across the posterior distribution of 10,000 trees using the ace function in *ape* v5.4-1 package^27^. For the ER model, all transitions rates were governed by a single parameter; for the SYM model, transitions 0 → 1 and 1 → 0 occur at the same rate but may differ from the transition rate between state 1 and 2; and in ARD model, each rate referred to a different parameter. Model fits were evaluated using the *fitDiscrete* function in the R package *geiger* v2.07^28^. Model fits were determined using the AIC values (Akaike Information Criterion) and AIC weight (AIC.w)^29–32^. The comparison between different transition rate models revealed that the equal rate (ER) model was rejected in favour of a more parametrised all rates different (ARD) model for rictal, lower rictal and interramal bristle presence while the ER model was selected for the narial bristle presence (Table 1). The *densitymap* function (*phytools*) was used to plot the consensus tree on to which the posterior density of rictal bristle presence/absence was mapped. The mapped value represented the probability of having rictal bristles present. Adobe Photoshop CC 2018 software (Adobe Systems Incorporated, San Jose, California) was used to customise the resulting radial tree.

**Table 1:** Comparison between the different transition rate models using AIC (Akaike Information Criterion) values and weight: equal-rates (ER), symmetrical (SYM) and all-rates-different (ARD) models for categorical data and Brownian motion (BM), Ornstein–Uhlenbeck (OU) and Early-burst (EB) models for continuous data.

Character	Model	AIC values	AIC weight
Rictal bristle presence	ER	455.2	0.00025
	SYM	455.2	0.00025
	**ARD**	438.6	**0.99950**
Lower rictal bristle presence	ER	673.8	0
	SYM	673.8	0
	**ARD**	581.2	**1**
Narial bristle presence	**ER**	516.4	**0.41388**
	SYM	516.4	0.41388
	ARD	518.1	0.17225
Interramal bristle presence	ER	573.2	0.00019
	SYM	573.2	0.00019
	**ARD**	556.1	**0.99962**

Values in bold illustrate the best fit model selected for the analysis.

### Sensitivity analysis

To check the robustness of our analyses to the trait used (bristle presence) and examine the uncertainty of our ancestral reconstruction for the presence of each facial bristle, we calculated the phylogenetic signals of each trait and evaluated both taxonomic sampling bias and tree.

#### Phylogenetic signals

We examined the phylogenetic signals to determine whether the distribution of each trait across our phylogeny follows a Brownian motion evolution hypothesis or shows divergent evolutionary trends suggesting potential selective pressures acting on our trait. We used Pagel’s λ^33^ for rictal bristle presence character (binary traits), using the *phylosig* function (package *phytools*)^25^, to evaluate its phylogenetic signal; a lambda value (λ) close to 1 and *P* < 0.05 signifies that the trait evolution would be predicted by Brownian motion model and a lambda value (λ) of 0 would signifies that the character has no phylogenetic signal.

#### Sampling bias

The robustness of the ancestral state reconstruction findings for facial bristle presence was evaluated by exploring the effects of sampling bias of the taxa and trees within the datasets for each *make.simmap* analysis. To evaluate taxonomic sampling bias, we calculated a probability of oversampled or undersampled families (number of representative of a family in the dataset divided by the total number of species known to exist in the family) to weight the likelihood of a given bird species appearing in a downsampled dataset (e.g.^34^). The probability was subsequently used in the weighted analysis by downsampling the datasets (full dataset, and presence only subset) to 70%, 80% and 90% of the species, preferentially removing families that are overrepresented. The stochastic mapping procedure was then repeated 10,000 times, with the mean number of transitions, gains and losses calculated, as well as the average time the character spent in each state, for each of the subsamples (70, 80, 90%) (Table 2).

**Table 2:** Sensitivity analysis results for rictal bristle presence: mapping of the stochastic character on 100 trees randomly sampled from the posterior distribution over 100 simulations, and mapping of the stochastic character with each weighted downsampled subsets (70%, 80%, 90%).

Analysis		Rictal bristle presence	Lower rictus	Nares	Interramus
Random trees	Average of changes	65	127	76	94
	Gains/Losses	15/50	6/121	53/23	14/80
	Time spent as absent	62%	65%	75%	62%
	Ancestral character state	Present	Present	Absent	Present
	Confidence in ASR	90%	99%	98%	99%
Subset 90%	Average of changes	55	119	72	82
	Gains/losses	18/37	3/116	50/22	19/63
	Time spent as absent	61%	66%	71%	60%
	Ancestral character state	Present	Present	Absent	Present
	Confidence in ASR	70%	100%	98%	98%
Subset 80%	Average of changes	50	105	64	73
	Gains/losses	14/36	4/101	46/17	18/55
	Time spent as absent	61%	67%	71%	62%
	Ancestral character state	Present	Present	Absent	Present
	Confidence in ASR	85%	100%	98%	96%
Subset 70%	Average of changes	51	93	52	75
	Gains/losses	17/34	8/85	37/15	4/71
	Time spent as absent	60%	66%	73%	56%
	Ancestral character state	Present	Present	Absent	Present
	Confidence in ASR	79%	99%	97%	100%

#### Tree topology uncertainty

We explored the uncertainty of tree topology and branch length in the ancestral state reconstruction analysis by randomly sampling 100 trees from the posterior distribution, in addition to the consensus tree. For each of these 100 trees, 100 character histories were randomly sampled, generating 10,000 character maps to account for the uncertainty associated with tree topology, branch length and timing of the transitions between morphological states (Table 2). We also tested if alternative phylogenetic topologies for Palaeognathae and Caprimulgimorphae, which would match recent studies (e.g.^13,35^), would have an effect on the ancestral state reconstruction analysis for facial bristle presence (Table S2), and obtained similar results to the analysis with our original consensus tree (Table S1, S2). 

#### Ecological traits

Species-specific ecological traits were added to the dataset using birdsoftheworld.org^14^, and included the following trait variables: (i) period of activity, (ii) habitat type, (iii) foraging method, (iv) foraging height, and (v) diet (Table 3). Instances where some species belonged to more than one dietary guild, diet categories were based on a combination of maximum two dietary guilds—the first guild (a; Table 3) corresponding to the main food type and the second guild (b, c, d; Table 3) corresponding to the secondary food type of a species (e.g. a–b, a–c, a–d, etc.). A total of 22 combinations were generated in our dataset for diet, e.g. Invertivore–Granivore, Invertivore–Vertivore and Invertivore–Frugivore, or Vertivore–Invertivore, Frugivore–Granivore or Frugivore–Herbivore, etc. Similarly, in instances where species exhibited more than one foraging method, the foraging method category was based on a combination of the two main foraging method. A total of 4 combinations were generated for the foraging method category, i.e. Gleaning–Hawking or Gleaning–Sallying, Hawking–Sallying, and Sallying–Plunge-diving.

**Table 3:** Ecological traits used in this study with their definitions and combinations.

Traits	Description
**Activity period**	
Diurnal	Diurnal species that forage during the day
Diurnal cathemeral	Predominantly diurnal species that occasionally foraged at dawn, dusk or even during the night
Nocturnal cathemeral	Predominantly nocturnal species that occasionally feed during daytime from late morning to late afternoon (partially diurnal)
Crepuscular	Forages at dusk and dawn and full moonlight night
Near obligate nocturnal	Forages both crepuscularly and nocturnally
Obligate nocturnal	Forages exclusively during the night
**Habitat type**	
Open	Scarcely wooded or bare area (e.g. grasslands, heathlands, clearings, wetlands, marshlands, scrublands, savannahs, desert, arid, semi-arid)
Semi-open	Loosely wooded area that is a mixture of open country and woodlands (e.g. corridors, woodlands, wooded savannahs, rangelands, riparian woodlands)
Closed	High, densely wooded areas (e.g. rainforests, tropical lowlands, subtropical montane forests)
**Diet**	
Invertivore	Feeds on flying insects and/or terrestrial invertebrates
Vertivore	Feeds on terrestrial vertebrates
Aquatic invertivore	Feeds on aquatic invertebrates (e.g. crustaceans and water-borne insects)
Aquatic vertivore	Feeds on aquatic vertebrates (e.g. fishes)
Aquatic herbivore	Feeds on aquatic vegetation (e.g. seaweed and algae)
Frugivore	Feeds on fruits
Granivore	Feeds on seeds
Nectarivore	Feeds on plant nectar and plant exudates
Herbivores	Feeds on vegetation parts (e.g. leaves, buds and flowers)
Omnivore	Feeds on invertebrates, vertebrates and plant material and exudates
**Foraging method**	
Skimming	Feeds along the surface of the water to capture prey
Dabbling	Immerses its head, neck and upper body while swimming to get submerged vegetation
Dipping	Briefly submerges itself, partially or completely, to obtain food
Diving	Submerges itself completely under water and swims to forage on vegetation or pursues prey (e.g. fishes, crustaceans)
Gleaning	Forages by pecking/picking meticulously food from nearby surfaces, such as tree bark, branch, leaves or grass and ground, without full extension of neck or legs and with no acrobatic movements involved
Hawking	Snatches food on the wing, without beginning from a perch and consuming the prey without perching
Sallying	Flies out from a perch to catch a prey in the air and returns to the perch
Lunging	Darts rapidly on prey using rapid leg movements rather than flight to approach and capture the prey, and often pauses between hunting strikes
Scratching	Dislodges section of substrate (dirt/debris from the ground) with foot movements to expose seeds or bugs
Plunge diving	Plunges into the water from a height to catch prey under the surface
Probing	Inserts its beak into a crevices or holes in firm substrates, or directly into soft substrates to extract hidden food
Scanning	Carefully watches over an area, either perched, hovering or soaring, before launching its attack to the ground or in the water
**Foraging height**	
Low	Forages on the ground and in understorey
Various	Forages at all levels from the ground to the canopy
High	Forages high within and above the canopy

### Model construction for bristle association with ecological traits

The same dataset of 1022 avian species and the consensus phylogenetic tree was used for this analysis. However, for this investigation, the rictal bristle length was calculated per individual (the average length of the rictal bristles measured per individual), rather than using a mean value per species.

To determine the relationship of both rictal bristle presence and length with species-specific ecological traits, a phylogenetically controlled Markov chain Monte Carlo generalised linear mixed model (MCMCglmm) was conducted, using the R package “MCMCglmm”^36^ in RStudio^23^. For rictal bristle presence, a binomial ‘threshold’ model was used to account for the binary response variable, whereas a “Gaussian” model was used for the rictal bristle length continuous response variable. In both models, the period of activity, the habitat type, the foraging method, the foraging height and diet were included as fixed effects. In both models, phylogeny and individual ID were included as independent random effects.

Following Hadfield^36^, a weak informative inverse-Gamma prior was used in the models, with variance (V) set to 1, and the belief parameter (nu) set to 0.002 for both the random effects structure (G-structure) and residual structure (R-structure). Residual variance was fixed in the absence of this information for the rictal bristle presence model since this used binary data (as per Hadfield, 2010^36^). Other parameter combinations were systematically explored but the models did not converge with them. The model was run for 800,000 iterations, with a burn-in period of 80,000, and a thinning of 40, which were determined using diagnostics in the coda package^37^. Three independent MCMC chains were run per model to check for model convergence using Gelman-Rubin diagnostics, with model convergence confirmed when the potential scale reduction factor required value was < 1.1^38^. Effective sample sizes (> 200) and autocorrelation (*P* < 0.05) values between successive iterations were also examined. Non-significant fixed effects (*p*MCMC > 0.10) were permanently excluded from the model formula if, in doing so, the fit of the model improved. Using ggplot2 package^39^, we constructed caterpillar plots representing the mean parameter estimates and the 95% credible intervals (CI) for each model. If the credible intervals were found to exclude zero, the parameter was considered significant with the model *P*-value given by the *p*MCMC value.

It was not possible to obtain a converging model for bristle shape since this was a categorical variable that exhibited a large range in the number of species in each category i.e. unbranched bristle shape was found in 292 species, while the branched shape was only found in 47 species, and branched at the base found to be present in 26 species. While visual inspection of MCMC chains suggested convergence after 12.8 million iterations, convergence was not supported by the Gelman–Rubin statistic; thus, a model for bristle shape was not considered further.

Following model construction and validation, a suitable ‘*reference category’* was selected for pairwise comparisons. These reference categories are compared to all others categories within their ecological traits, and tested for significant differences^40^. Since all Anatidae species recorded in our dataset did not have rictal bristles and shared the same ecological categories, these categories were selected as reference categories for the rictal bristle presence and length models. Therefore, the reference categories were diurnal, open, dabbling, low and aquatic herbivore.

## Supplementary Information

Supplementary Table S1.

## Data Availability

All data analysed during this study are included in the published article and its supplementary information file. The dataset generated during the current study is available in the figshare repository, 10.6084/m9.figshare.20486256.
