# Supplementary material for: The evolutionary origin of avian facial bristles and the likely role of rictal bristles in feeding ecology
Source: Sci Rep. 2022 Dec 6;12:21108. doi: 10.1038/s41598-022-24781-7 (PMC9726833; doi:10.1038/s41598-022-24781-7)
Supplement: Supplementary file 1 — Supplementary Table S1. [file 41598_2022_24781_MOESM1_ESM.pdf]

The evolutionary origin of avian facial bristles and the likely role of rictal bristles in feeding ecology

Mariane G Delaunay[1*], Charlotte Brassey[1], Carl Larsen[2], Huw Lloyd[1], Robyn A Grant[1]

1. Department of Natural Sciences, Manchester Metropolitan University, Manchester, M1 5GD UK
2. School of Life Sciences, University of Liverpool, Liverpool, UK

Supplementary material

Table S1. **Ancestral state reconstruction** (ASR) **of the stochastic character facial bristle presence for each facial bristle type and normalised rictal bristle length**: lower rictal, narial and interramal, with their phylogenetic signal on to the phylogeny.

Facial bristles	Average n of changes	Gain	Loss	Phylogenetic Signal	Ancestral character state	Confidence in ASR	Time spent as absent
**Rictal**	63	16	47	$\lambda=0.89$ $P<0.001$	Present	87%	61%
**Lower rictal**	127	3	124	$\lambda=0.73$ $P<0.001$	Present	99.9%	66.8%
**Narial**	74	52	22	$\lambda=0.95$ $P<0.001$	Absent	98.4%	71.61%
**Interramal**	87	23	64	$\lambda=0.89$ $P<0.001$	Present	96.8%	60.9%

Table S2. Ancestral reconstruction (ASR) **of the stochastic character facial bristles for each facial bristle type, using a modified consensus tree for Palaeognathae and Caprimulgimorphae tree topologies.** The modified tree was obtained by manually moving branches of our original consensus tree (at family level) in Mesquite, in order to match tree topologies found in recent studies (e.g. Yonezawa *et al.*, 2017; Chen *et al.*, 2019).

Facial bristles	Average n of changes	Gain	Loss	Ancestral character state	Confidence in ASR	Time spent as absent
**Rictal**	63	16	47	Present	98%	60%
**Lower rictal**	127	3	124	Present	100%	67%
**Narial**	77	55	21	Absent	97%	73%
**Interramal**	97	5	91	Present	100%	58%
